# Supplementary material for: Causal Relationship between Aging and Anorexia Nervosa: A White-Matter-Microstructure-Mediated Mendelian Randomization Analysis
Source: Biomedicines. 2024 Aug 16;12(8):1874. doi: 10.3390/biomedicines12081874 (PMC11351342; doi:10.3390/biomedicines12081874)
Supplement: Supplementary file 1 [file biomedicines-12-01874-s001.zip › Supplementary Figure/Figure S4/Singe SNP plot of MR analysis for TL to ubm-b-2023.pdf]

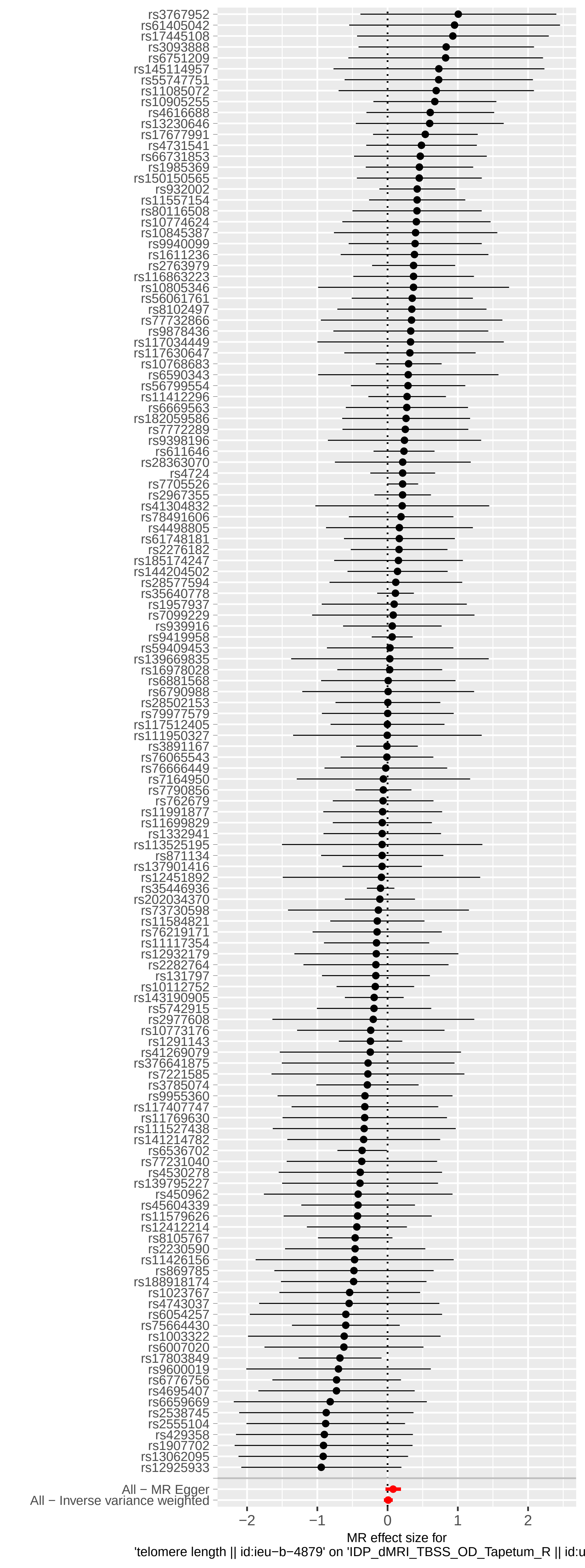
rs3767952
rs61405042
rs17445108
rs3093888
rs6751209
rs145114957
rs55747751
rs11085072
rs10905255
rs4616688
rs13230646
rs17677991
rs4731541
rs66731853
rs1985369
rs150150565
rs932002
rs11557154
rs80116508
rs10774624
rs10845387
rs9940099
rs1611236
rs2763979
rs116863223
rs10805346
rs56061761
rs8102497
rs77732866
rs9878436
rs117034449
rs117630647
rs10768683
rs6590343
rs56799554
rs11412296
rs6669563
rs182059586
rs7772289
rs9398196
rs611646
rs28363070
rs4724
rs7705526
rs2967355
rs41304832
rs78491606
rs4498805
rs61748181
rs2276182
rs185174247
rs144204502
rs28577594
rs35640778
rs1957937
rs7099229
rs939916
rs9419958
rs59409453
rs139669835
rs16978028
rs6881568
rs6790988
rs28502153
rs79977579
rs117512405
rs111950327
rs3891167
rs76065543
rs76666449
rs7164950
rs7790856
rs762679
rs11991877
rs11699829
rs1332941
rs113525195
rs871134
rs137901416
rs12451892
rs35446936
rs202034370
rs73730598
rs11584821
rs76219171
rs11117354
rs12932179
rs2282764
rs131797
rs10112752
rs143190905
rs5742915
rs2977608
rs10773176
rs1291143
rs41269079
rs376641875
rs7221585
rs3785074
rs9955360
rs117407747
rs11769630
rs111527438
rs141214782
rs6536702
rs77231040
rs4530278
rs139795227
rs450962
rs45604339
rs11579626
rs12412214
rs8105767
rs2230590
rs11426156
rs869785
rs188918174
rs1023767
rs4743037
rs6054257
rs75664430
rs1003322
rs6007020
rs17803849
rs9600019
rs6776756
rs4695407
rs6659669
rs2538745
rs2555104
rs429358
rs1907702
rs13062095
rs12925933
All − MR Egger
All − Inverse variance weighted
−2
−1
0
1
2
MR effect size for
'telomere length || id:ieu−b−4879' on 'IDP_dMRI_TBSS_OD_Tapetum_R || id:u
